# Beach sand oil spills select for generalist microbial populations

**DOI:** 10.1038/s41396-021-01017-6

**Published:** 2021-06-04

**Authors:** Patrick Heritier-Robbins, Smruthi Karthikeyan, Janet K. Hatt, Minjae Kim, Markus Huettel, Joel E. Kostka, Konstantinos T. Konstantinidis, Luis M. Rodriguez-R

**Affiliations:** 1 School of Civil and Environmental Engineering, Georgia Institute of Technology, Atlanta, GA USA; 2 Department of Earth, Ocean and Atmospheric Sciences, Florida State University, Tallahassee, FL USA; 3 School of Biological Sciences, Georgia Institute of Technology, Atlanta, GA USA; 4 School of Earth and Atmospheric Sciences, Georgia Institute of Technology, Atlanta, GA USA; 5 Center for Microbial Dynamics and Infection, Georgia Institute of Technology, Atlanta, GA USA; 6 Department of Microbiology, University of Innsbruck, Innsbruck, Austria; 7 Digital Science Center (DiSC), University of Innsbruck, Innsbruck, Austria

**Keywords:** Microbial ecology, Community ecology

## Abstract

The specialization-disturbance hypothesis predicts that, in the event of a disturbance, generalists are favored, while specialists are selected against. This hypothesis has not been rigorously tested in microbial systems and it remains unclear to what extent it could explain microbial community succession patterns following perturbations. Previous field observations of Pensacola Beach sands that were impacted by the Deepwater Horizon (DWH) oil spill provided evidence in support of the specialization-disturbance hypothesis. However, ecological drift as well as uncounted environmental fluctuations (*e.g*., storms) could not be ruled out as confounding factors driving these field results. In this study, the specialization-disturbance hypothesis was tested on beach sands, disturbed by DWH crude oil, ex situ in closed laboratory advective-flow chambers that mimic in situ conditions in saturated beach sediments. The chambers were inoculated with weathered DWH oil and unamended chambers served as controls. The time series of shotgun metagenomic and 16S rRNA gene amplicon sequence data from a two-month long incubation showed that functional diversity significantly increased while taxonomic diversity significantly declined, indicating a decrease in specialist taxa. Thus, results from this laboratory study corroborate field observations, providing verification that the specialization-disturbance hypothesis can explain microbial succession patterns in crude oil impacted beach sands.

## Introduction

Deciphering the role of disturbance in microbial community dynamics is key to understanding ecosystem resilience and recovery. The specialization-disturbance hypothesis, coined by Vázquez and Simberloff [1], predicts that disturbances negatively affect specialists while benefiting generalists. This is because specialist taxa are adapted to narrow niches, and thus are selected against when disturbance transforms their environment outside of their niche boundaries. In contrast, generalists are thought to have larger genomes and encode for a larger number of (distinct) gene functions, which could facilitate adaptation immediately after disturbances [2, 3]. Although several previous studies applied ecological theory to describe the response and recovery of community dynamics to disturbance [4, 5], the relationship of disturbance and specialization remains largely unexplored for microbial systems [6]. Specifically, disturbed communities are often observed to encompass reduced taxonomic and/or phylogenetic diversity compared with undisturbed controls, but whether this pattern also translates to increased functional diversity remains largely unknown. We recently presented support for the specialization-disturbance hypothesis when we showed that reduced taxonomic diversity coupled to increased functional diversity characterized the response of sedimentary microbial communities from Pensacola Beach (Florida, USA) to the Deepwater Horizon (DWH) crude oil spill [7].

Specifically, weathered oil from the DWH well blowout washed ashore between June and July 2010, with Total Petroleum Hydrocarbon (TPH) concentrations reaching up to ~2 kg per m of beach. Waves and storms buried the oil and mixed it with the beach sand, forming a migratory oiled sand layer in the sediment column [8]. Shotgun metagenomic and 16S rRNA gene amplicon sequencing of beach sand samples revealed distinct successional patterns where generalist hydrocarbon degrading taxa such as *Alcanivorax* and “*Candidatus* Macondimonas” rapidly increased while specialist taxa such as the chemolithoautotrophic archaea *Nitrosopumilus* substantially deceased in abundance [7, 9, 10]. However, the open-system nature of the field study meant that dispersal mechanisms had potential effects on the derived results that could not be accounted for, including the possibility that functionally diverse taxa were transported to the sampling site. Therefore, whether or not the increase in functional diversity associated with oil spill taxa necessarily demonstrated a larger niche breadth remained to be tested.

**Fig. 1:**
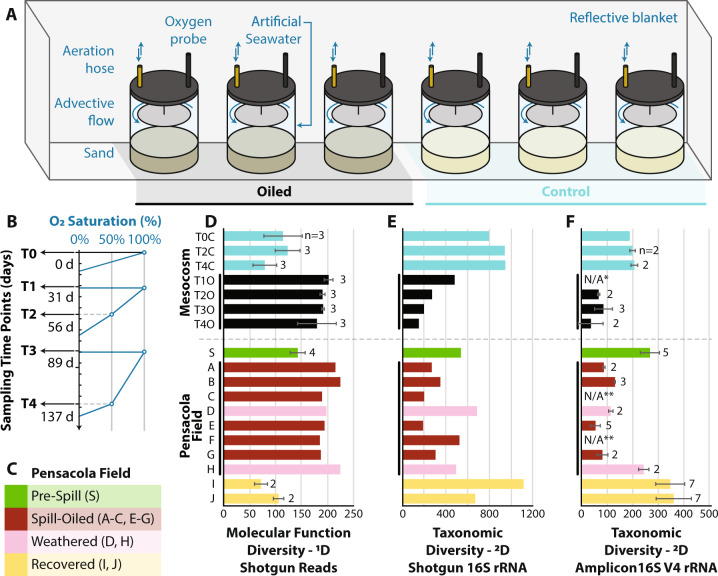
Experimental design and diversity results. The ex situ mesocosm experiment (**A**) tested the effect of oxic-anoxic cycles (**B**) on microbial oil biodegradation. The mesocosm experiment had three oiled chambers and three control chambers that were sampled at specific oxygen saturation points (oxygen levels dropped due to microbial oil consumption and were reestablished based on manual aeration of the chambers). Additionally, datasets from a field study at Pensacola Beach (**C**) that had samples taken before oil contaminated the beach (Pre-Spill), while the beach was contaminated with oil (Spill-Oiled, and Weathered), and after oil had been removed/biodegraded (Recovered) were reanalyzed here. Molecular functional diversity was evaluated with the Chao-Shen Entropy Estimator and converted to true diversity (^1^D) to be in effective GO terms (**D**). Taxonomic diversity based on metagenomic 16S rRNA gene fragments (**E**) and 16S rRNA gene V4 amplicons (**F**) was evaluated with coverage-based rarefaction at diversity order *q* = 2. ^2^D is equivalent to the inverse Simpson index and represents the effective number of dominant OTUs. In **D** through **F**, Pensacola field samples are designated by letters that are identical to those used in our previous study [7] and shown in **C**. In categories with replicates, the sample means are shown with one standard deviation and the number of replicates analyzed. N/A* indicates that no DNA from T1O amplified during PCR. N/A** indicates that **C** and **F** did not have amplicons sequenced for them.

In the present study, these concerns were addressed by using a closed laboratory mesocosm system with a homogenous oil disturbance that eliminated dispersal mechanisms (Figs. 1 and S1). We employed advective transport chambers that closely simulate redox oscillations and water transport phenomena observed in the sandy beach intertidal zone [11] (Fig. 1) and allowed for consistent hydrocarbon degradation dynamics (Fig. S2). Consistent with these expectations, we observed a high degree of similarity between the microbial communities in the chambers when inoculated with DWH oil and the previous field samples [12], further validating our mesocosm as tools to reliably study in situ microbial processes. For the present study, generalist taxa were defined as operational taxonomic units (OTU) having broad niche breadth reflected by increased number of non-redundant protein functions (measured as the number of molecular function gene-ontology -GO- terms), whereas specialist taxa were defined as OTUs having a narrow niche breadth characterized by fewer non-redundant protein functions.

## Results and discussion

We performed shotgun metagenome (assessing functional diversity) and 16S rRNA gene V4 amplicon (assessing taxonomic diversity) sequencing of time-series samples from the closed laboratory mesocosm chambers with oil addition (oiled) or without (control) to test whether or not the specialization disturbance hypothesis could explain the microbial community succession patterns (response). Additionally, metagenomic datasets from the Pensacola Beach field study [4] were included for comparison. The latter datasets represented beach sands before the oil had reached the coast (Pre-Spill), while the beach was contaminated (Spill-Oiled and Weathered), and after the oil concentrations in beach sands had reached undetectable levels (Recovered) (Fig. 1). The detailed description of the sample processing, sequencing, and bioinformatic analyses can be found in the Supplementary Online material (Figs. S3 and S4). Nonpareil, a tool that estimates what fraction of the microbial community is represented in a metagenome (i.e., the coverage) by examining the level of redundancy among the metagenomic reads [13], showed that coverage of the sampled microbial communities by sequencing was adequate for comparison [14], with 60–75% sample coverage for oiled mesocosm and 45–70% for control sample. In addition, Nonpareil sequence diversity (*N*_*d*_), an estimate of the total diversity in sequence space harbored by a microbial community, and other diversity metrics showed that control samples (no oil added) harbored higher diversity. Applying the commonly used pipeline of assigning 16S rRNA gene fragments recovered in the metagenomes against the SILVA database release 132 (ref. [15]) using VSEARCH in QIIME2 [16] and 97% nucleotide identity for a match (closed OTU picking) resulted in 11% fewer reads assigned for control *vs*. oiled mesocosm metagenomes and 27% fewer reads assigned for clean *vs*. oiled Pensacola metagenomes.

These results indicated that the control samples potentially harbored more novel (uncharacterized) taxa that could confound taxonomic comparisons due to the comparatively lower number of taxonomically identified sequences. To account for this effect, we employed a manual pipeline with BLASTn [17], and a lower cut-off (90% nucleotide identity) for read assignment to the database (Method 2). Additionally, we performed our analysis based on both 16S rRNA gene amplicon sequences as well as 16S-carrying metagenomic reads, and employed Hill numbers, represented as ^*q*^*D*, a group of diversity indices that take into account species abundance and richness to compute the equivalent number of species at an order *q*, where *q* adjusts the sensitivity to rare species (see also [18] and references therein). Our results, after rarefying the 16S rRNA gene fragment OTU abundance to the metagenomic dataset with the lowest coverage [18], showed that the inverse Simpson index (^2^D) was lower in oiled chambers with a mean of 274 (SD = 146) than in control chambers with a mean of 896 (SD = 86; Table 1). The Welch’s *t*-test revealed a significant difference at alpha 0.05 (*p* value = 8.89e−4). Amplicon data from the same mesocosm samples (Fig. 1) or analysis at the sequence variant level (ASVs; Fig. S5) showed similar results (Fig. 1). See supplementary results and discussion for further details (Fig S6).

**Table 1:** Summary statistics and hypothesis testing for functional diversity and species diversity indices.

Mesocosm Experiment									
	Oiled				Control				Welch *p* value
	Mean	SD	*n*	AD *p* value	Mean	SD	*n*	A-D *p* value	
Molecular function diversity (^1^D)	191	18.4	12	0.000630	106	31.9	9	0.353	1.14 × 10^−5^
Taxonomic diversity (^2^D) 16 S	274	146	4	NA	896	85.8	3	NA	8.89 × 10^−4^
Taxonomic diversity (^2^D) V4	67.3	35.8	7	NA	201	11.3	5	NA	1.50 × 10^−5^

Pensacola field study									
	Oiled				Control				Welch *p* value
	Mean	SD	*n*	AD *p* value	Mean	SD	*n*	A-D *p* value	
Molecular function diversity (^1^D)	199	16.5	6	NA	116	33.4	8	0.770	7.28 × 10^−5^
Taxonomic diversity (^2^D) 16S	304	124	6	NA	775	304	3	NA	0.123
Taxonomic diversity (^2^D) V4	84.7	34.2	12	0.502	332	67.3	19	0.529	8.54 × 10^−14^

^1^D is the true diversity of molecular functions in effective GO terms. ^2^D, equivalent to the inverse Simpson index, is the effective number of dominant OTUs. SD is the standard deviation. AD *p* value is the *p* value for Anderson Darling normality test. The AD *p* values were not computed when *n* < 8. The two-tailed Welch’s *t*-test was considered significant when the *p* value was <0.05.

Functional diversity was analyzed based on the number of metagenomic reads matching molecular function gene ontology (GO) terms [19] as previously described [7]. Our analysis showed that functional diversity (^1^D) was higher in oiled chambers with a mean of 193 equivalent GO terms (SD = 18) compared to control chambers with a mean of 105 equivalent GO terms (SD = 31; Table 1; Chao-Shen Entropy Estimator; *p* value = 1.14e-05, two-tailed Welch’s t-test).

Collectively, the results presented here from closed system mesocosms, which were designed to limit fluctuations in environmental conditions, stochasticity, and dispersal, showed that the specialization disturbance hypothesis can explain microbial succession patterns following crude oil perturbations in coastal beach sand environments. Recent incubation experiments of microbial communities from sandy soils have also provided evidence in support of the specialization-disturbance hypothesis (preprint available at the time of writing [6]), and the close agreement of these results with those of previous field observations (e.g., Table 1, Fig. 1) [7] suggested that this underlying explanation/mechanism is robust even in light of environmental variation and drift. The high similarity in taxonomic composition between our mesocosms and our previous field data also suggested that the key oil degraders were present in the clean sands at the time of sampling for establishing the mesocosms, 7 years after the DWH oil spill. The survival strategy of oil degraders in the clean sand remains an interesting question, and has implications for whether or not their niche breadth includes uncontaminated sandy sediments. It would be interesting to test whether similar patterns are observed in other habitats (e.g., beach sand from an alternative source lacking a history of oil exposure) and other types of perturbation in order to test the universal applicability of the results reported here. With sufficient background data available on the unperturbed ecosystem, we believe that the approach outlined here based on specialist *vs*. generalist taxa should be able to elucidate whether or not the specialization disturbance can explain microbial responses to other types of perturbations and/or identify recovered ecosystems.

## Supplementary information

Supplementary Online Material

## Data Availability

All sequencing data is available through the NCBI Sequence Read Archive (SRA) under BioProject identifiers PRJNA722673 (mesocosm experiment amplicons), PRJNA690167 (mesocosm metagenomes), and PRJNA260285 (field experiment). Additional metadata is available through the Gulf of Mexico Research Initiative Information & Data Cooperative (GRIIDC) at https://data.gulfresearchinitiative.org/data/R5.x278.000:0001.

## References

[1] Vázquez DP, Simberloff D (2002). Ecological specialization and susceptibility to disturbance: conjectures and refutations. Am Nat.

[2] Konstantinidis KT, Tiedje JM (2004). Trends between gene content and genome size in prokaryotic species with larger genomes. Proc Natl Acad Sci.

[3] Martiny JBH, Jones SE, Lennon JT, Martiny AC (2015). Microbiomes in light of traits: a phylogenetic perspective. Science.

[4] Prosser JI, Bohannan BJM, Curtis TP, Ellis RJ, Firestone MK, Freckleton RP (2007). The role of ecological theory in microbial ecology. Nat Rev Microbiol.

[5] Shade A, Peter H, Allison SD, Baho DL, Berga M, Bürgmann H (2012). Fundamentals of microbial community resistance and resilience. Front Microbiol.

[6] Chen Y-J, Leung PM, Bay SK, Hugenholtz P, Kessler AJ, Shelley G, et al. Metabolic flexibility allows generalist bacteria to become dominant in a frequently disturbed ecosystem. bioRxiv. 2020; 2020.02.12.945220. 10.1038/s41396-021-00988-w PMC8443593 33941890

[7] Rodriguez-R LM, Overholt WA, Hagan C, Huettel M, Kostka JE, Konstantinidis KT (2015). Microbial community successional patterns in beach sands impacted by the Deepwater Horizon oil spill. ISME J.

[8] Huettel M, Overholt WA, Kostka JE, Hagan C, Kaba J, Wells WB (2018). Degradation of Deepwater Horizon oil buried in a Florida beach influenced by tidal pumping. Mar Pollut Bull.

[9] Karthikeyan S, Rodriguez-R LM, Heritier-Robbins P, Kim M, Overholt WA, Gaby JC (2019). “*Candidatus* Macondimonas diazotrophica”, a novel gammaproteobacterial genus dominating crude-oil-contaminated coastal sediments. ISME J.

[10] Kostka JE, Prakash O, Overholt WA, Green SJ, Freyer G, Canion A (2011). Hydrocarbon-degrading bacteria and the bacterial community response in gulf of mexico beach sands impacted by the deepwater horizon oil spill. Appl Environ Microbiol.

[11] Huettel M, Rusch A (2000). Transport and degradation of phytoplankton in permeable sediment. Limnol Oceanogr.

[12] Karthikeyan S, Kim M, Heritier-Robbins P, Hatt JK, Spain JC, Overholt WA (2020). Integrated omics elucidate the mechanisms driving the rapid biodegradation of deepwater horizon oil in intertidal sediments undergoing oxic–anoxic cycles. Environ Sci Technol.

[13] Rodriguez-R LM, Gunturu S, Tiedje JM, Cole JR, Konstantinidis KT (2018). Nonpareil 3: fast estimation of metagenomic coverage and sequence diversity. mSystems.

[14] Rodriguez-R LM, Konstantinidis KT (2014). Estimating coverage in metagenomic data sets and why it matters. ISME J.

[15] Quast C, Pruesse E, Yilmaz P, Gerken J, Schweer T, Yarza P (2012). The SILVA ribosomal RNA gene database project: improved data processing and web-based tools. Nucleic Acids Res.

[16] Bolyen E, Rideout JR, Dillon MR, Bokulich NA, Abnet CC, Al-Ghalith GA (2019). Reproducible, interactive, scalable and extensible microbiome data science using QIIME 2. Nat Biotechnol.

[17] Camacho C, Coulouris G, Avagyan V, Ma N, Papadopoulos J, Bealer K (2009). BLAST+: architecture and applications. BMC Bioinforma.

[18] Hsieh TC, Ma KH, Chao A (2016). iNEXT: an R package for rarefaction and extrapolation of species diversity (Hill numbers). Methods Ecol Evol.

[19] Ashburner M, Ball CA, Blake JA, Botstein D, Butler H, Cherry JM (2000). Gene ontology: tool for the unification of biology. Nat Genet.

