# Supplementary material for: Beach sand oil spills select for generalist microbial populations
Source: ISME J. 2021 Jun 4;15(11):3418–22. doi: 10.1038/s41396-021-01017-6 (PMC8528907; doi:10.1038/s41396-021-01017-6)
Supplement: Supplementary file 1 — Supplementary Online Material [file 41396_2021_1017_MOESM1_ESM.pdf]

Supplemental Text

Materials and Methods

Mesocosm chamber setup

Beach sand was collected from the intertidal zone at Pensacola Beach (FL, USA), PB (30°119.57 N, 87°110.47 W) and incubated in six advection flow mesocosm chambers (Figure S1) [1, 2]. Each chamber received 2 kg of sand (bulk mass + moisture mass). In three of the chambers weathered Macondo oil was mixed in at a concentration of 5 mg/g of sand. The three other chambers served as controls (no oil added). Each chamber was then filled with 22 ppt salinity artificial seawater (Instant Ocean® sea salt in DI water). The chambers were placed inside a large tank, which was also filled with artificial seawater to reduce the effects of laboratory temperature fluctuations. Chambers were equipped with online dissolved oxygen sensors (PreSens GmBH, Regensburg, Germany) for continuous oxygen and temperature monitoring. An air pump connected to the chambers allowed aeration of the water column at certain time points (see below). The entire experimental setup was covered with an opaque tarp to block light from reaching the chambers and prevent photosynthesis.

Mesocosm sampling timeline

Samples were taken at five time points (0, 31, 56, 89, and 137 days) and are referred to in chronological order as T0, T1, T2, T3, and T4. All chambers were aerated to saturation before T0 and after T1 and T3. This allowed T0, T2, and T4 to be sampled under oxic conditions and T1 and T3 to be sampled under anoxic conditions. Anoxic conditions developed through the microbial respiration of oil. Hence, cycles of oxic-anoxic conditions were established with anoxic phases of 12-15 days. This setting simulates the lower intertidal section of sandy beaches where flushing with oxygen may only occur at the ebb tide of spring tides (*i.e.*, at approximately 14-day intervals), and prolonged anoxic conditions are not uncommon. At each time point not all chambers were sampled. At T0 only control chambers were sampled, and at T2 and T4 only oiled cambers were sampled. At T1 and T3 all chambers were sampled. Panel B of Figure 1 displays the sampling timeline and its relation to the oxygen saturation level of the chambers.

Sample collection and processing

At each time point, glass corers were used to sample the sands. Corers were constructed by removing the tips from glass pipettes and beveling the cut edges. During sampling, a corer was pushed into the sand until it reached the chamber bottom. Multiple locations were sampled in each chamber to reduce sample error due to possible heterogeneity from uneven oil distribution. Sampled sands were flash frozen with liquid nitrogen and then stored at -80 ℃ until processing. Ammonia, nitrite, nitrate, and sulfate were measured in collected porewater using a Hach spectrophotometer (Hach Instruments, Loveland, CO, USA). Hydrocarbon and TPH fingerprinting and moisture analyses were carried out at Eurofins Lancaster Laboratories (Lancaster, PA, USA).

DNA extraction and sequencing

Community DNA was extracted from samples using the DNeasy PowerSoil kit (Qiagen Inc., Germantown, MD, USA). DNA concentrations were quantified using the Qubit 2.0 fluorometer with Qubit HS DNA kit (ThermoFisher Scientific, Waltham, MA, USA). DNA libraries were prepared using the Illumina Nextera XT DNA library prep kit according to manufacturer's

instructions except the protocol was terminated after isolation of cleaned double stranded libraries. Average insert sizes of DNA libraries and concentrations were determined on a Bioanalyzer 2100 instrument (Agilent, Santa Clara, CA, USA). An equimolar pool of the sequencing libraries was sequenced on an Illumina HiSeq 2500 instrument (School of Biological Sciences, Georgia Institute of Technology) using the HiSeq Rapid PE Cluster Kit v2 and HiSeq Rapid SBS Kit v2 (Illumina) for 300 cycles (2 x 150 bp paired end). Adapter trimming and demultiplexing of sequenced samples were carried out by the HiSeq 2500 instrument.

When possible, remaining DNA was used to amplify the V4 region of the 16S rRNA gene. Template DNA at variable concentration, Accuprime Pfx polymerase at 0.5 U, Accuprime 10X buffer at 1X, and forward and reverse primers at 200 nM each, were added to molecular grade water (final concentrations given). The Accuprime 10X buffer contained deoxyribonucleotide triphosphates (dNTPs) and $MgSO_4$ diluted to a final concentration of 0.3 mM and 1 mM, respectively. Primers used are described by Kozich et al [3], and result in ~385 bp sequences (~250 bp of the 16S V4 region + 135 bp of primer). DNA was amplified for 25 cycles with denaturation at 95 °C for 30 seconds, annealing at 55 °C for 30 seconds and extension at 72 °C for 60 seconds. Amplified DNA was cleaned with SPRIselect beads (Beckman Coulter, Brea, CA, USA) following the left side size selection protocol of the manufacturer with a 0.75 ratio of beads to sample. DNA concentrations were measured with the Qubit HS DNA kit and average insert size was measured with the Bioanalyzer 2100 instrument. Libraries were prepared and then sequenced on the Illumina MiSeq platform for 500 cycles (2 x 250 bp PE).

Pensacola Beach field study samples

In addition to the mesocosm experiment samples, which are being analyzed for the first time here in the context of the specialization disturbance hypothesis, sequence data from the Pensacola Beach field study is reanalyzed [18]. The Pensacola Beach field study samples were taken during and after the time of the Deepwater Horizon oil spill. The first set of samples labeled pre and S were taken before oil had reach the beach during May of 2010 (pre spill). Oil contamination of the beach began early in June. The second set of samples, labeled A through D, were taken from the oil contaminated beach sands at the end of July in 2010 (oiled). The third set samples, labeled E through H, were taken from the oil contaminated beach sands in October 2010 (oiled). Samples D and H contained weathered oil distinguishing them from the other oiled samples. The final set of samples labeled I and J, were collected in June of 2011 from sands where oil was undetectable (recovered).

Quality control and 16S rRNA gene (16S) fragment recovery from metagenomes and amplicons

Sequenced 16S V4 amplicon paired end reads were merged using PEAR (v0.9.0.) [4]. Shotgun metagenome reads and merged amplicon reads were trimmed and quality checked using SolexaQA++ (v3.1.3) [5]. Reads were trimmed with a PHRED score cutoff of 20 (>99% accuracy per base-position). Only trimmed reads greater than 100 bp and 50 bp were retained for V4 amplicon and shotgun metagenome reads, respectively. Additionally, 16S rRNA gene carrying short reads were recovered from shotgun metagenomes with Metaxa2 (v2.2) [6]. Only reads identified as bacterial by Metaxa2 were used for downstream taxonomic diversity analysis.

Genome size and 16S rRNA gene copy number estimation
Reads encoding the single copy ribosomal RNA polymerase subunit B gene (*rpoB*) were identified in the metagenomic samples with ROCker (v1.2.0) using the precomputed 100 bp *rpoB* model [7]. The sequencing depth of the *rpoB* gene was calculated by summating the alignment length of all matching reads, divided by the gene length. The 16S rRNA gene copy number was calculated by dividing the total number of base pairs of 16S rRNA gene-carrying reads by the product of the *rpoB* sequencing depth and the length of the 16S rRNA gene (taken here as 1,522 bp). The average genome size for each sample was calculated by dividing the total metagenomic base pairs by the *rpoB* sequencing depth.

OTU clustering
Three different methods (see below) were tested to cluster 16S rRNA gene carrying reads into OTUs. The first two methods were chosen to cluster non-overlapping reads carrying different parts (fragments) of the 16S rRNA gene. Since reads were non-overlapping, these two methods used a closed reference picking approach. The third method was used only for the overlapping 16S rRNA gene amplicons (V4 variable region) so it used an open reference picking approach. All three methods used the SILVA-132 database (pre-clustered at 99% identity) as the reference database [8].

Method 1, which used q2-vsearch, represents a common approach for this purpose. This method typically applies a high nucleotide identity cutoff (*i.e.*, 97%), but early results showed at a high cutoff, the percentage of reads being assigned to clusters was greatly reduced in some samples, especially the control mesocosm or clean field samples. We suspected that novel taxa not represented in the database had a higher relative abundance in clean samples versus oiled samples causing the lower frequency of reads assigned to a database 16S rRNA gene reference sequence. To deal with this, we used a low nucleotide cutoff (90%) to capture reads from novel taxa as part of Method 1; we also tested a second method (Method 2) as described below.

Method 2 employed BLASTn and applied the same 90% nucleotide identity cutoff as described above for Method 1, but with an additional step. This step refined the OTUs by clustering their (matching) representative reference sequences at a lower nucleotide identity level (>90% identity) than the reference database had originally been clustered at (99% identity). This refinement reduced the number of OTUs and made their taxonomic identification possible only at a higher level (higher than genus).

To determine which of the first two methods performed better, the fraction of 16S rRNA gene fragments assigned to OTUs was calculated, and the mean and standard deviation of this metric were compared between the methods. It is important to note that this metric is unaffected by the CD-HIT clustering step of the reference sequences in Method 2, since this step only reduces the number of OTUs, not the number of reads assigned to OTUs.

Method 3 is a standard approach for overlapping 16S rRNA gene amplicons (V4 variable region), which assigns all reads and circumvents biases in the reference database (open OTU picking).

Method 1. Reads were dereplicated and closed reference clustering was performed with q2-vsearch in QIIME 2 [9] based on a minimum threshold of 90% identity for assigning a read to a reference sequence/OTU.

Method 2. Reads were queried with BLASTn (v2.2.29) [10] against the reference database. These results were filtered for the best match and only matches above 90% identity and 80% alignment length were used for further analysis. After filtering, the full-length 16S rRNA reference sequences for all assigned query reads were clustered to 97% identify with CD-HIT (v4.6.1) [11]. When reference sequences clustered, the number of reads from the filtered BLASTn results matching each of the references sequences were summated and assigned to the representative reference sequence of the cluster.

Method 3. Reads were dereplicated and open reference clustering was performed at 98.5% identity with q2-vsearch in QIIME 2.

Amplicon Sequence Variant (ASV) detection for 16S V4 amplicons

In addition to the OTU clustering the was performed with Method 3 as described above, the DADA2 pipeline was also used to detect amplicon sequence variants (ASVs) in the 16S V4 rRNA sequence dataset [21]. DADA2 was run on each sequencing run separately and the results were merged into single ASV table for analysis. For each sequencing run samples were pooled to increase the dada method's sensitivity to detect rare variants.

Taxonomic diversity analysis

Abundance tables from OTU clustering Method 2 and Method 3 were used for taxonomic diversity analysis. Method 2 performed better than Method 1; consequently, Method 1 results were excluded from further analysis. The abundance data from biological replicates were summed for Method 2 to ensure sufficient coverage. Singletons and doubletons were not removed to avoid altering coverage-based statistics. Taxonomic diversity was estimated based on coverage rarefaction of Hill numbers (^0D, ^1D, ^2D) using the R package iNEXT (v2.0.19) [12]. Taxonomic diversity was also analyzed based on ASVs results using iNEXT.

Functional diversity analysis

The abundance of shotgun metagenomic reads assigned to molecular function gene-ontology (GO) terms [13] served as the basis to assess functional diversity shifts across time and treatments. Metagenomic short reads were first assembled with IDBA-UD [14] using default parameters and gene-encoding regions were identified on the resulting contigs with MetaGeneMark [15]. Genes were translated to amino acid sequences and queried against the Swiss-Prot database [16] using BLASTp for functional annotation prediction. BLAST results were filtered for the best match for each query and for matches above 60% amino-acid identity and 60% alignment length. The matching Swiss-Prot references were mapped to GO terms and filtered for molecular functions. To determine the number of observed reads matching GO terms, BLASTn was used to query short reads against the genes, which had matched a GO term. BLASTn results were filtered for the best match of each query and then for matches with the product of alignment length and identity above 95%.

The Chao-Shen estimate of Shannon entropy H [17] for molecular functions was calculated in the R package entropy from the observed read counts of the molecular function GO terms. This

estimation uses adjustments for missing species (here GO terms) and sample coverage. The exponential of the estimated Shannon entropy was taken to covert the statistic to true diversity (^1D) with units of effective GO terms.

Hypothesis Testing

Samples were grouped categorically as oiled or clean to test the impact of the oil disturbance. For the mesocosm experiment samples, control samples labeled as T#C# formed the clean group and oiled samples labeled as T#O# formed the oiled group. For the Pensacola field study pre-spill samples (labeled pre/S) and recovered samples (labeled I and J) formed the clean group; and impacted samples (labeled A, B, C, E, F, and G) formed the oiled group, consistent with our previous field study [18]. If the group sample size was large enough (n > 7) the Anderson-Darling test from the R package nortest (v1.0-4) [19] was used to test if the data followed a normal distribution. A two-tailed t-test assuming unequal variances (Welch's t-test) was applied to compare the mean of the species diversity and functional diversity across groups.

Metagenome and amplicon samples labeled D and H were excluded from statistical tests because they could not be clearly distinguished as oiled or clean. Metagenomes OS-D and OS-H were both assigned an oil qualitative score of 0 (clean). However, when all metagenomes were compared by mash distances, OS-D grouped with other oiled metagenomes, OS-F, OS-E, and OS-G, which all had an oil qualitative score of 3. Metagenome OS-H did not group with any samples and appeared between the previously mentioned grouping and a grouping of the recovered samples (OS-J604, OS-598, OS-I600, and OS-I604) [20]. Importantly, even though OS-H was assigned an oil qualitative score of 0, its DNA was pooled from samples with different oil qualitative scores: OS-334 (score of 3) and OS-335 (score of 0).

Results

Sequencing Metrics

Shotgun metagenomic sequencing was performed on community DNA extracted from sediments. The number of trimmed reads after QC can be found in the supplemental Excel file (Sheet: Metagenome Read Metrics). Metaxa2 was used to recover 16S rRNA gene fragments from shotgun metagenome reads. Bacterial 16S rRNA genes accounted for 91% and 84% of the total recovered 16S rRNA gene sequences for all mesocosm and field samples, respectively (Figure S3). Only bacterial 16S rRNA gene fragments were used for OTU analysis. Coverage of recovered 16S rRNA gene fragments is discussed below with the taxonomic diversity analysis.

OTU Clustering

Two closed-reference methods were compared for clustering the recovered 16S rRNA gene fragments. De novo and open reference methods are known to be ineffective for shotgun data since recovered fragments originate from different sections of the 16S rRNA gene commonly with no overlap. Method 1 used VSEARCH in QIIME2 and Method 2 used BLASTn and CD-HIT. The second method was developed as a compromise between discarding reads from novel bacteria and effectively clustering OTUs at the genus/species level. The performance of the methods was compared by evaluating the fraction of bacterial 16S rRNA gene reads that were assigned to an OTU for each sample (Figure S4). The fraction of reads assigned was lower for Method 1 with a mean of 0.9066 (SD = 0.1381) compared to Method 2 with a mean of 0.9381

(SD = 0.0303). Pre-spill samples (OS-S1 to OS-S4) from Pensacola Beach were outliers for both methods, but had significantly fewer reads assigned with Method 1 than Method 2.

Based on these results, Method 2 was chosen for the closed reference OTU clustering of the 16S rRNA gene fragments. Method 2 resulted in a total of 26,965 SILVA reference representatives once the BLASTn results for all samples were filtered. These representatives were clustered with CD-HIT at 97% ID to form 20,714 OTUs. Across all samples, 35.60% of the OTUs had one observation (singletons) and 15.88% had two observations.

Clustering of the reference sequences at lower nucleotide identity (97%) was used in Method 2 to account for the fact that the assignment of the reads to a reference (close OTU picking) was performed at lower nucleotide identity (90%), and thus required a coarser definition of OTUs for a more direct comparison across samples. However, excluding this step did not provide for different results (data not shown but available by the authors upon request).

Amplicon reads were clustered with an open reference approach using VSEARCH in QIIME2 forming 49,061 OTUs, of which 15.86% were SILVA references and 84.14% were de-novo. Across all samples, 46.92% of OTUs had one observation (singletons) and 14.09% had two observations.

Taxonomic Diversity Analysis

Taxonomic diversity was assessed based on recovered 16S rRNA gene fragments from shotgun metagenomes and V4 amplicons. Hill numbers (^0D, ^1D, and ^2D) were used to quantify taxonomic diversity, which were made comparable between samples by using coverage-based rarefaction. For this, the sample with the minimum coverage was used to interpolate the Hill numbers for other samples; for the 16S rRNA gene fragment analysis the pooled reads from metagenomes S1, S2, S3, and S4 had the minimum coverage (71.5%), and for the V4 amplicon analysis sample OS-J598 had the minimum coverage (71.6%). Shifts in taxonomic diversity for Hill numbers ^0D, and ^1D are shown in Figure S6.

For the ASV results sample OS.579 (I group) had the minimum sample coverage at 94.6%. We believe DADA2 methods elimination of singletons may artificially increase the coverage as computed by iNEXT. Therefore, we plot the taxonomic diversity results from the ASV analysis separately in Figure S5.

Supplemental Figures

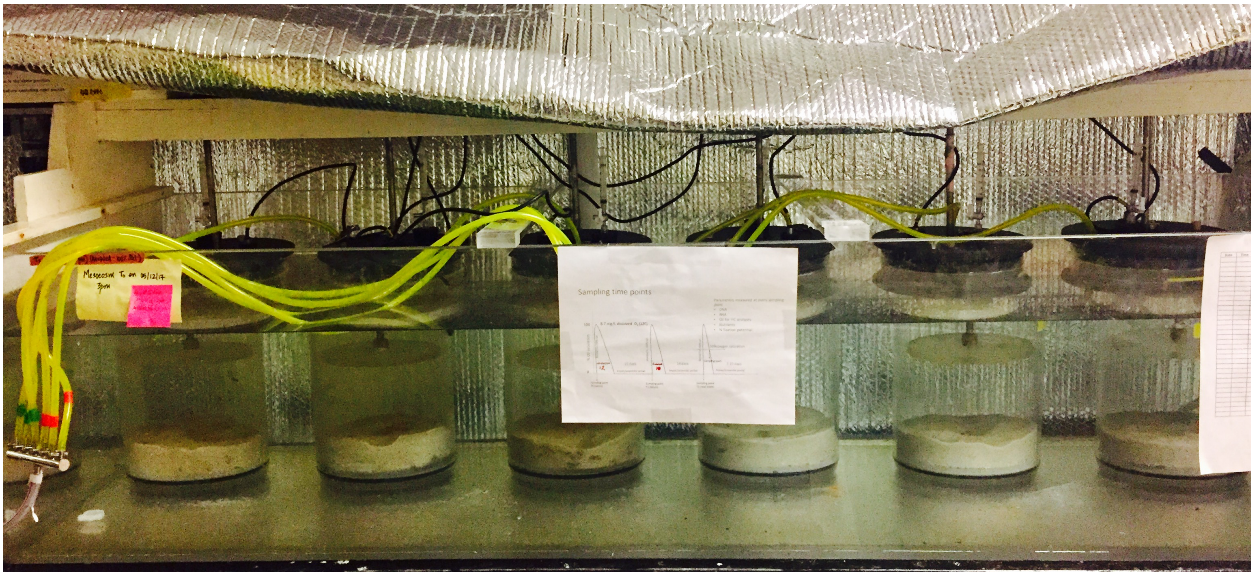

Figure S1: Physical mesocosm chamber configuration. The six mesocosm chambers filled with sand and artificial seawater sat in a large water-filled tank for thermal control. The three leftmost chambers were enriched with oil and the three rightmost chambers served as controls. The top of the each chambers was sealed to be airtight, with ports for the yellow aeration hose, oxygen probe (black wires), and disk shaft. A single motor rotated the disks seen inside each chamber at a constant speed forming an advection flow. The reflective blanket seen in the background covered the entire setup in order to prevent photosynthesis from occurring in the chambers. The cover and chamber tops were only open at sampling time points.

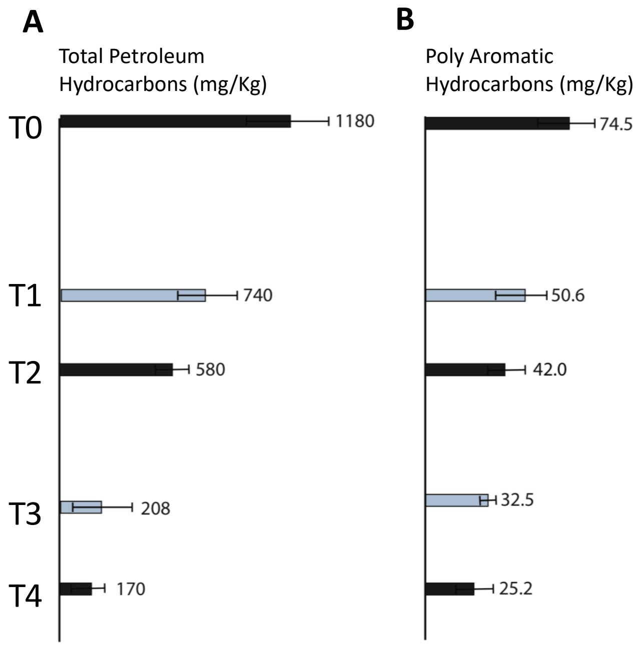

Figure S2: Mesocosm oil analysis. This figure is adapted from Karthikeyan et al. [20] with the permission of the authors and journal. Panel A and B display the total petroleum hydrocarbon (TPH) and polycyclic aromatic hydrocarbons (PAH) concentration in the oiled chambers, respectively. Concentrations are based on chemical analysis and are calculated in terms of the dry sand mass.

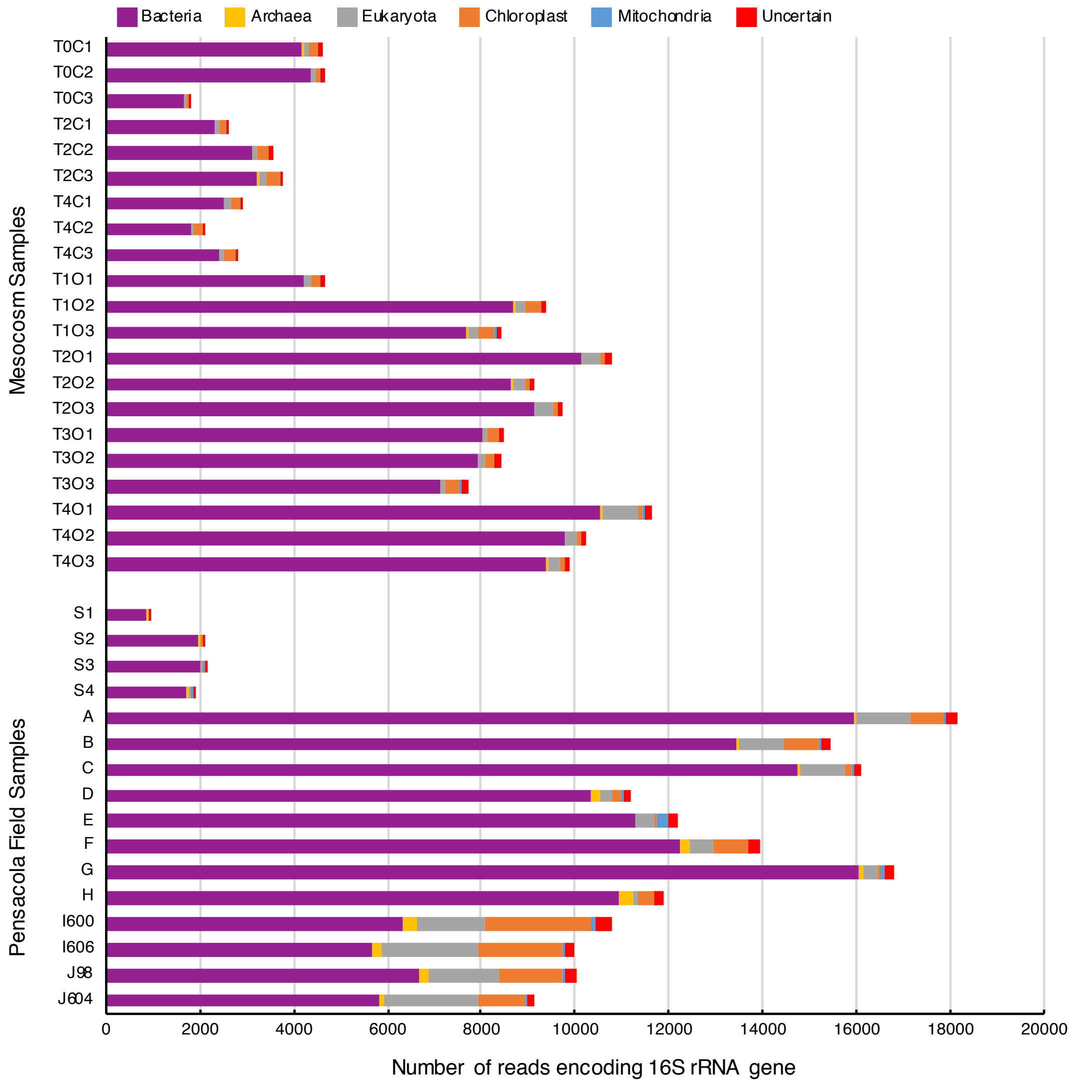

Figure S3: Recovery of SSU rRNA gene fragments from metagenomes with Metaxa2. Metaxa2 was used to recover metagenomic short reads carrying fragments of SSU rRNA genes. Reads were classified as originating from Bacteria, Archaea, Eukaryota, Chloroplasts, Mitochondria or uncertain. Only reads classified as carrying bacterial 16S rRNA gene fragments (purple) were used for downstream taxonomic diversity assessment.

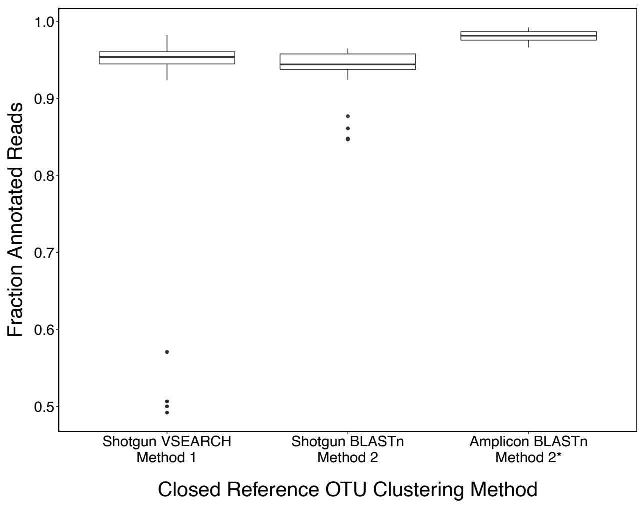

Figure S4: Comparison of VSEARCH and BLASTn to perform closed reference OTU clustering of non-overlapping reads. For each method the boxplot shows the distribution of the fraction of annotated reads for all samples. The fraction of annotated reads is the fraction of bacterial 16S rRNA gene-carrying reads assigned to OTU (y-axis) out of total 16S rRNA gene-carrying reads found by Metaxa2 under the respective methods (x-axis). For the shotgun 16S rRNA gene fragments, both methods show the four pre-spill samples as outliers (low fractions). Since Method 2 had a lower variance and higher mean it was selected for OTU clustering in the analysis. The third boxplot benchmarks how Method 2 performed when it was used with the 16S rRNA gene V4 amplicons.

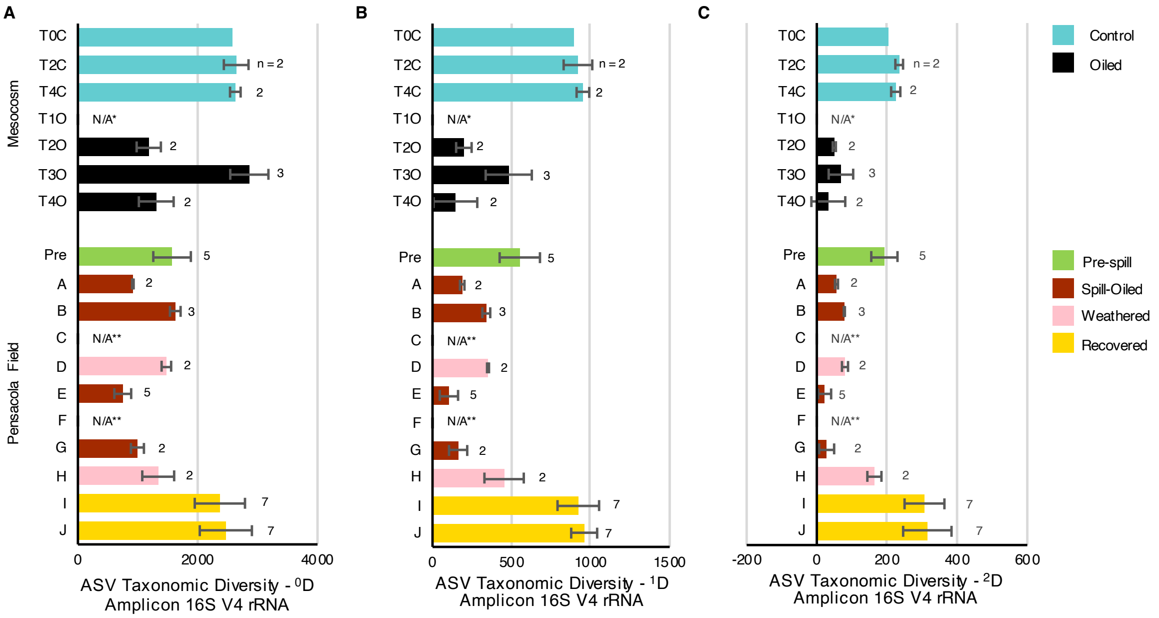

Figure S5: Taxonomic diversity using ASV approach for 16S V4 rRNA

Taxonomic diversity was evaluated at Hill numbers ^{0}D (A) and ^{1}D (B) and ^{2}D (C) for ASVs. N/A* indicates that no DNA from T1O amplified during PCR. N/A** indicates that C and F did not have amplicons sequenced for them.

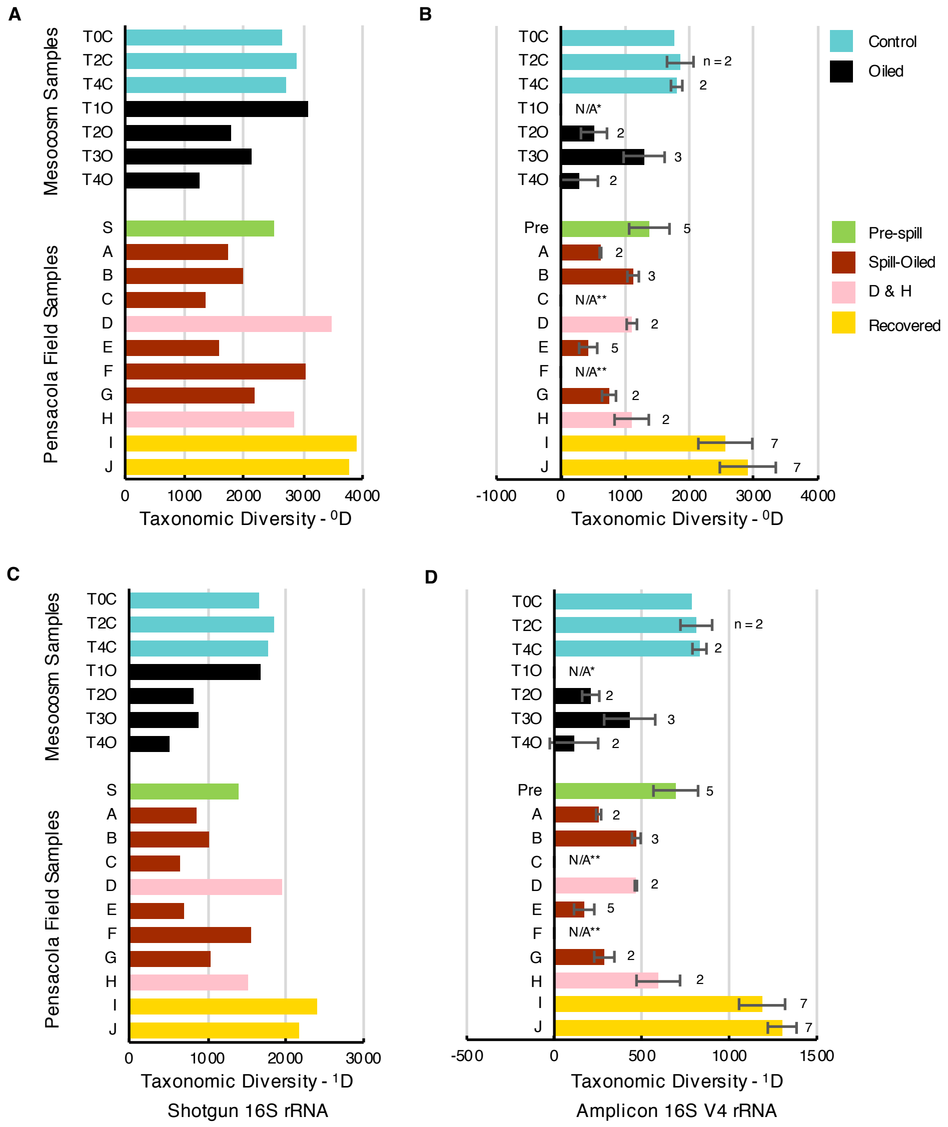

Figure S6: Comparison of taxonomic diversity at lower orders. Taxonomic diversity was evaluated at multiple Hill numbers ^0D (top) and ^1D (bottom) for metagenomic 16S rRNA gene fragments (left) and 16S rRNA gene V4 amplicons (right). N/A* indicates that no DNA from T1O amplified during PCR. N/A** indicates that C and F did not have amplicons sequenced for them.

References

1. Khalili A, Basu AJ, Huettel M. A non-Darcy model for recirculating flow through a fluid-sediment interface in a cylindrical container. *Acta Mech* 1997; **123**: 75–87.
2. Janssen F, Faerber P, Huettel M, Meyer V, Witte U. Pore-water advection and solute fluxes in permeable marine sediments (I): Calibration and performance of the novel benthic chamber system Sandy. *Limnol Oceanogr* 2005; **50**: 768–778.
3. Kozich JJ, Westcott SL, Baxter NT, Highlander SK, Schloss PD. Development of a Dual-Index Sequencing Strategy and Curation Pipeline for Analyzing Amplicon Sequence Data on the MiSeq Illumina Sequencing Platform. *Appl Environ Microbiol* 2013; **79**: 5112–5120.
4. Zhang J, Kobert K, Flouri T, Stamatakis A. PEAR: a fast and accurate Illumina Paired-End reAd mergeR. *Bioinformatics* 2014; **30**: 614–620.
5. Cox MP, Peterson DA, Biggs PJ. SolexaQA: At-a-glance quality assessment of Illumina second-generation sequencing data. *BMC Bioinformatics* 2010; **11**: 485.
6. Bengtsson-Palme J, Hartmann M, Eriksson KM, Pal C, Thorell K, Larsson DGJ, *et al*. metaxa2: improved identification and taxonomic classification of small and large subunit rRNA in metagenomic data. *Mol Ecol Resour* 2015; **15**: 1403–1414.
7. Orellana LH, Rodriguez-R LM, Konstantinidis KT. ROCker: accurate detection and quantification of target genes in short-read metagenomic data sets by modeling sliding-window bitscores. *Nucleic Acids Res* 2017; **45**: e14.
8. Quast C, Pruesse E, Yilmaz P, Gerken J, Schweer T, Yarza P, *et al*. The SILVA ribosomal RNA gene database project: improved data processing and web-based tools. *Nucleic Acids Res* 2012; **41**: D590–D596.

9. Bolyen E, Rideout JR, Dillon MR, Bokulich NA, Abnet CC, Al-Ghalith GA, *et al*. Reproducible, interactive, scalable and extensible microbiome data science using QIIME 2. *Nat Biotechnol* 2019; **37**: 852–857.

10. Camacho C, Coulouris G, Avagyan V, Ma N, Papadopoulos J, Bealer K, *et al*. BLAST+: architecture and applications. *BMC Bioinformatics* 2009; **10**: 421.

11. Li W, Godzik A. Cd-hit: a fast program for clustering and comparing large sets of protein or nucleotide sequences. *Bioinformatics* 2006; **22**: 1658–1659.

12. Hsieh TC, Ma KH, Chao A. iNEXT: an R package for rarefaction and extrapolation of species diversity (Hill numbers). *Methods Ecol Evol* 2016; **7**: 1451–1456.

13. Ashburner M, Ball CA, Blake JA, Botstein D, Butler H, Cherry JM, *et al*. Gene ontology: tool for the unification of biology. The Gene Ontology Consortium. *Nat Genet* 2000; **25**: 25–29.

14. Peng Y, Leung HCM, Yiu SM, Chin FYL. IDBA-UD: a de novo assembler for single-cell and metagenomic sequencing data with highly uneven depth. *Bioinformatics* 2012; **28**: 1420–1428.

15. Zhu W, Lomsadze A, Borodovsky M. Ab initio gene identification in metagenomic sequences. *Nucleic Acids Res* 2010; **38**: e132.

16. The UniProt Consortium. UniProt: a worldwide hub of protein knowledge. *Nucleic Acids Res* 2019; **47**: D506–D515.

17. Chao A, Shen T-J. Nonparametric estimation of Shannon's index of diversity when there are unseen species in sample. *Environ Ecol Stat* 2003; **10**: 429–443.

18. Rodriguez-R LM, Overholt WA, Hagan C, Huettel M, Kostka JE, Konstantinidis KT. Microbial community successional patterns in beach sands impacted by the Deepwater Horizon oil spill. *ISME J* 2015; **9**: 1928–1940.

19. Gross J, Ligges U. nortest: Tests for Normality. 2015.

20. Karthikeyan S, Kim M, Heritier-Robbins P, Hatt JK, Spain JC, Overholt WA, *et al*. Integrated Omics Elucidate the Mechanisms Driving the Rapid Biodegradation of Deepwater Horizon Oil in Intertidal Sediments Undergoing Oxic–Anoxic Cycles. *Environ Sci Technol* 2020; **54**: 10088–10099.

21. Callahan, B, McMurdie, P, Rosen, M, *et al.* DADA2: High-resolution sample inference from Illumina amplicon data. *Nat Methods* 2016; **13:** 581–583.
